# Exploring Successful Parameter Region for Coarse-Grained Simulation of Biomolecules by Bayesian Optimization and Active Learning

**DOI:** 10.3390/biom10030482

**Published:** 2020-03-21

**Authors:** Ryo Kanada, Atsushi Tokuhisa, Koji Tsuda, Yasushi Okuno, Kei Terayama

**Affiliations:** 1 RIKEN Compass to Healthy Life Research Complex Program, Kobe 650-0047, Japan; tokuhisa@riken.jp (A.T.); okuno.yasushi.4c@kyoto-u.ac.jp (Y.O.); 2 RIKEN Center for Computational Science, Kobe 650-0047, Japan; 3 RIKEN Medical Sciences Innovation Hub Program, Yokohama 230-0045, Japan; 4 Graduate School of Frontier Sciences, he UTniversity of Tokyo, Kashiwa 277-8561, Japan; tsuda@k.u-tokyo.ac.jp; 5 RIKEN Center for Advanced Intelligence Project, Tokyo 103-0027, Japan; 6 Research and Services Division of Materials Data and Integrated System, National Institute for Materials Science, Tsukuba 305-0047, Japan; 7 Graduate School of Medicine, Kyoto University, Kyoto 606-8507, Japan

**Keywords:** coarse-grained molecular dynamics simulation, biological rotary motor, machine learning, active learning, bayesian optimization

## Abstract

Accompanied with an increase of revealed biomolecular structures owing to advancements in structural biology, the molecular dynamics (MD) approach, especially coarse-grained (CG) MD suitable for macromolecules, is becoming increasingly important for elucidating their dynamics and behavior. In fact, CG-MD simulation has succeeded in qualitatively reproducing numerous biological processes for various biomolecules such as conformational changes and protein folding with reasonable calculation costs. However, CG-MD simulations strongly depend on various parameters, and selecting an appropriate parameter set is necessary to reproduce a particular biological process. Because exhaustive examination of all candidate parameters is inefficient, it is important to identify successful parameters. Furthermore, the successful region, in which the desired process is reproducible, is essential for describing the detailed mechanics of functional processes and environmental sensitivity and robustness. We propose an efficient search method for identifying the successful region by using two machine learning techniques, Bayesian optimization and active learning. We evaluated its performance using F1-ATPase, a biological rotary motor, with CG-MD simulations. We successfully identified the successful region with lower computational costs (12.3% in the best case) without sacrificing accuracy compared to exhaustive search. This method can accelerate not only parameter search but also biological discussion of the detailed mechanics of functional processes and environmental sensitivity based on MD simulation studies.

## 1. Introduction

Owing to significant advancements in molecular structural analysis such as electron cryo-microscopy (Cryo-EM) and X-ray free-electron laser (XFEL), thousands of three-dimensional biomolecular structures including macromolecules and complex systems have been revealed. Furthermore, with the progress of molecular dynamics (MD) simulations using GPUs and supercomputers, it has become possible to understand the dynamics and behavior of biomolecular systems. Although all-atom molecular dynamics (AA-MD) simulation is a powerful tool for studying the dynamics of a biomolecule, it remains difficult to simulate entire functional processes of large molecules such as membrane proteins and motor proteins whose typical time scales are around milliseconds or longer. For example, even using a special-purpose supercomputer dedicated to AA-MD simulations, the protein folding process can be simulated only for proteins that are small and have a relatively high folding speed [1]. To overcome this problem, the application of coarse-grained (CG) MD simulations has been attracting attention because of its lower calculation cost compared to that of AA-MD. In fact, CG-MD simulation has succeeded in qualitatively reproducing numerous biological processes for various biomolecules [2,3,4,5,6,7,8,9,10,11,12]. Especially, C${}_{\alpha }$ switching Go model [3,5] and multiple basin Go model [13] can realize large conformational change of protein easily by using available multiple native structures as references to construct model interaction.

However, CG-MD simulations strongly depend on the various model and environmental parameters and their tuning is needed to reproduce desired biological processes. In general, many CG-parameters are directly or indirectly influenced by the environment such as the temperature and ionic strength and mutation. Particularly, determining CG-parameters for a larger system with drastic conformational changes and interactions between multiple-chains is quite difficult and generally results in some uncertainty related to model parameters: for example, in the multiple-basin model [13] coupling parameter Δ and relative stability ΔV, in Langevin simulation of CG-model the friction constant Γ, inter-molecular interaction strength, parameter related to ion-strength dependence and so on. Various methods for determining these parameters such as force matching [14] and fluctuating matching [15] have been proposed. However, in many cases, these methods are very computationally expensive and cannot always determine valid parameters. As a result, such tuning is often performed manually.

Furthermore, to understand molecular mechanics through simulations, it is important to investigate the region of successful parameters (in other words, a phase diagram) that reproduce a targeted process. For example, a phase diagram of environmental parameters such as temperature, ionic strength and mutation is expected to provide information on the sensitivity or robustness to environmental changes and mutations of the target molecule. Particularly, the range of CG-parameters related to mutation may provide insights into the design new molecule with better functions. A few studies [4,16] have been conducted to systematically investigated and validated the dependence of the CG-model parameters by drawing a phase diagram and the limitation of CG-models have been discussed, including some parameter uncertainty.

However, exhaustively examining all candidate parameters is inefficient. Specifically, as simulations are performed stochastically, their results vary depending on a seed of the random variable and initial conditions of MD. To determine whether a process occurs stably (i.e., beyond a certain probability) under a certain parameter, it is necessary to repeatedly perform calculations while changing the conditions. As a result, the computational cost of exhaustively examining parameters with MD simulations is extremely high.

In recent years, various parameter optimization methods [17] such as Bayesian optimization (BO) and evolutionary algorithms have been proposed in the field of machine learning and applied to a wide range of actual problems such as parameter optimization of deep neural networks [18,19], combination of materials [20], and protein design [21]. Most parameter optimization techniques effectively find the optimal parameter. However, it is not necessarily appropriate to efficiently search for parameters beyond a certain criterion. In contrast, one of the authors recently proposed an effective sampling method [22,23] for constructing phase diagrams based on uncertainty sampling (US), a type of active learning technique. The method based on US can efficiently determine phases to examine the phase boundary preferentially when two or more phases are sampled. By regarding successful parameters and the failed parameters as two phases, it is possible to efficiently search for successful parameters. However, if the number of successful parameters is small, the efficiency of the method is considered to be poor, as boundary sampling becomes difficult because of difficulties in detecting successful parameters.

In this study, we propose a method named BOUS that efficiently samples in successful regions by combining BO and US described above to overcome the computational cost of parameter search in MD simulations. BOUS first searches for a successful parameter based on the success rate of the targeted process by using BO, and then switches to the US to efficiently search for successful parameter regions. To evaluate the performance of BOUS, we applied sampling methods including BOUS to parameter search problems of the rotational motion for F1-ATPase based on CG-MD simulations. We performed the CG-MD simulations based on two types of dynamics, Newtonian and Langevin. We also evaluated the sampling performances of other sampling methods: exhaustive search, random sampling (RS), US, and BO. The results showed that BOUS, BO, and US identified successful regions and construct a phase diagram with drastically reduced computation compared to exhaustive search and RS. In addition, BOUS showed better performances than BO and US.

Moreover, we confirmed that the rotational motion of the F1-motor was reproduced over a wide range containing parameters that were not reported in existing studies. We also discussed the stability information against parameter perturbation based on the constructed phase diagrams of successful parameters. These results suggest that deeper mechanical and biological discussions can be accelerated by efficiently drawing phase diagrams. Our implementation is available at https://github.com/tsudalab/SPEMD.

## 2. Materials and Methods

### 2.1. Machine Learning Based Region Search of Successful Parameters

**Figure 1:**
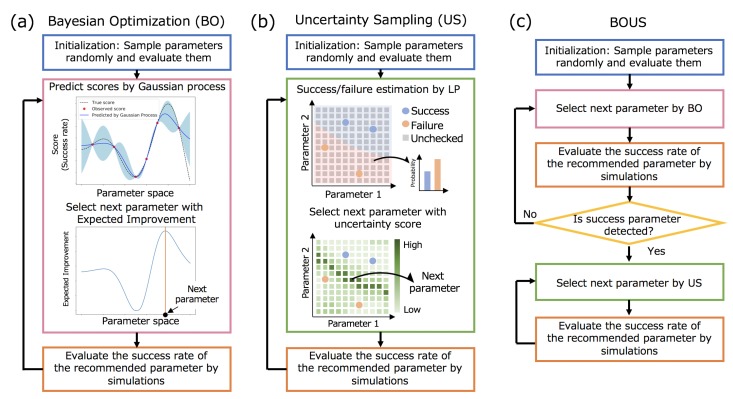
Flowcharts of efficient region search of successful parameters by using Bayesian optimization (**a**) and uncertainty sampling (**b**). (**c**) The combination of Bayesian optimization (BO) and uncertainty sampling (US) (BOUS) for successful parameter region search.

This study was conducted to effectively identify the region of successful parameters that stably realize the desired dynamics, e.g., rotation of the F1-motor, even if random conditions such as initial velocity distribution are altered. We refer to a parameter that achieved a certain success rate or more as a successful parameter in this paper. The detailed definition of the success rate for the rotation F1-motor is described in a later section. We propose a method named as BOUS for efficiently sampling all successful parameters in the parameter space by combining BO and US. Figure 1a–c show flowcharts of the sampling methods on the basis of BO, US, and BOUS. After explaining the approaches of BO and US, the details of BOUS are described.

#### 2.1.1. Parameter Sampling By Bo

Recently, as a type of data-driven approach, BO has been widely used as an efficient search method for parameters that satisfy a desired property [17,24]. As typical application examples, BO was used to efficiently search for the best network in deep learning [18] to optimize network parameters such as the number of networks and learning rates, and the best combination of a large number of materials [20].

Figure 1a shows the procedure used for a high score parameter search using BO. First, some parameters are selected randomly, and their scores are obtained by simulations as initialization. Next, the BO framework recommends a parameter to be examined next based on the obtained dataset of the checked parameters and their results, using a machine learning method called Gaussian process [24]. The expected mean and variance of the score for each parameter set are predicted by Gaussian process, as shown in the upper figure in Figure 1a. Then, the next parameter is selected by using an acquisition function, as shown in the lower figure in Figure 1a. Here, we used the expected improvement [25] as the acquisition function. Next, the simulation for the recommended parameter is performed and its score is added to the dataset. By repeating the process of a recommendation and simulation, we can efficiently search for the parameter with a desired property.

In BO, the score is basically assumed to be a quantitative value. In F1-motor simulation, we can apply the BO framework by regarding the success rate of its rotation as the score to efficiently find the parameter with the highest rate. However, BO may be inefficient for successful region search because BO is specialized for searching for parameters with higher scores.

In this study, we adopted the COMBO library [26] as an implementation of BO. COMBO is one of the accelerated implementation of BO using the approximation of the Gaussian process based on the random feature map [27]. This approach can be used without tuning of hyperparameters, as they are automatically determined by maximizing the type-II likelihood [24] in COMBO.

#### 2.1.2. Parameter Sampling by Us

An active learning-based approach proposed by some authors for efficiently constructing a phase diagram [22] is expected to be useful for identifying regions of successful parameters. The approach adopted uncertainty sampling, which is a type of active learning, for preferentially sampling around predicted boundaries of multiple discrete classes by machine learning to quickly determine the boundaries. In this study, we applied the US-based sampling framework to F1-motor simulations by regarding successful and failed parameters as two phases. However, when only one phase is identified, any boundary of the phase cannot be predicted, resulting in inefficient sampling behavior close to the random sampling. In this research, if the criterion of the success rate was high, the region of successful parameters was narrow, making it difficult to detect the successful parameters.

Figure 1b shows the procedure of successful region search based on US. We implemented the method based on a combination of the label propagation (LP) [28] and the least confidence method [29], which showed the best performance among algorithms described in previous studies [22,23]. We assigned a successful or failed label to each parameter based on a success rate. In the sampling procedure of US, we first predicted the probability distributions for all unexamined parameters using LP, as shown in the upper figure in Figure 1b. Next, the least confidence method was used to select the parameter with the largest uncertainty, as shown in the lower figure in Figure 1b. The uncertainty score for each parameter x is calculated as follows:

(1) $$\begin{array}{c} \begin{array}{c} u\left(x\right)=1-\max_{p}P\left(p|x\right) \end{array}, \end{array}$$

where *p* is a label (success or failure) and P(p|x) is the predicted probability of *p* of the parameter x. That is, if the predicted probabilities of success and failure are almost the same, the score is relatively high. We used default hyperparameters in the previous paper [22]. It was shown that US works well under default parameters.

#### 2.1.3. Combination of BO and US

Based on the advantages and disadvantages of BO and US described above, we propose the use of BOUS for searching for a successful parameter based on BO initially, and samples based on US to cover successful parameters after finding a successful parameter. Figure 1c shows the procedure of BOUS. Even if the region of the successful parameter is narrow, BOUS can efficiently find a successful parameter by BO. In the BOUS implementation, we use COMBO as a BO implementation with the success rate data to search until a successful parameter is found. After it is found, we assign successful or failed labels to the checked parameters according to the success rate and switch to the search based on US as described above.

### 2.2. F1-Motor and CG-MD Simulation

**Figure 2:**
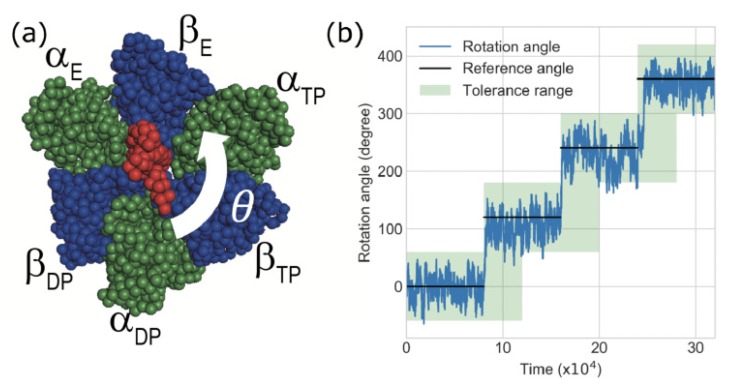
(**a**) Structure of F${}_{1}$-ATPase motor and counterclockwise rotation angle θ from initial structure. The red part corresponds to γ-subunit, and the blue and green parts correspond to the β and α subunits, respectively. (**b**) Example of time-series of the rotation angle θ. Black lines indicate the switched angle, referred to as the reference angle of the gamma-subunit in coarse-grained (CG)-molecular dynamics (MD) simulations. The light-green region shows the proper angle region, which is referred to as the tolerance angle. The goodness score of F1-motor rotation is defined as the ratio of the angle θs in the tolerance range.

F1-ATPase is a biological rotary motor that catalyzes the hydrolysis of ATP into ADP and Pi. As shown in Figure 2a, the F1-motor consists of seven subunits $\alpha_{3}\beta_{3}\gamma$ of which x-ray crystal structure (Abrahams et al., 1994 [30]) was obtained from the PDB entry of 1BMF. The central stalk γ is a rotor, while the alternately arranged $\alpha_{3}$ and $\beta_{3}$ subunits form a ring acting as the stator of the rotary motor. In x-ray structure nucleotide state of each subunit is classified three states: an ATP bound state “TP”, ADP-bound state “DP”, and nucleotide free state “E”. The γ-subunit rotates counterclockwise unidirectionally in discrete 120-degree steps as each ATP is hydrolyzed [31].

In this study, to conduct MD simulation for rotational motion of γ subunit in F1-motor by using CafeMol software [32] according to simulation scheme as shown in Figure S1. We applied structure-based C${}_{\alpha }$ switching Go model [3] in which one particle represents each amino acid in proteins. The total energy for the F1-motor consists of four terms: $V=V_{\gamma }+V_{\alpha_{3}\beta_{3}}+V_{\alpha_{3}\beta_{3}-\gamma }+V_{anchor}$. All parameters described by Koga’s work [3] were used, except for one related to excluded volume interaction (EVI) between γ and $\alpha_{3}\beta_{3}$ subunits: $V_{\alpha_{3}\beta_{3}-\gamma }$ is defined as

(2) $$\begin{array}{c} V_{\alpha_{3}\beta_{3}-\gamma }=\sum_{i\in \alpha_{3}\beta_{3},j\in \gamma }EVI\times \epsilon {\left(C/r_{ij}\right)}^{12}, \end{array}$$

where EVI is one of the targets evaluated in the parameter survey. See the supplemental text-S1 for the detailed information of potentials: $V_{\gamma }$, $V_{\alpha_{3}\beta_{3}}$, $V_{\alpha_{3}\beta_{3}-\gamma }$, $V_{anchor}$, and simulation protocol.

### 2.3. Simulation Dynamics and Sampled Parameter Space

To demonstrate that our optimization method is robust regardless of the type of dynamics of CG simulation and dimension of sampled parameter space, we simulated the time evolution of the CG F1-system with two representative dynamics: Newtonian dynamics and under-damped Langevin dynamics.

For time integration of Newton dynamics “velocity Verlet” algorithm [33] was used with a step size of dt=0.1. Time steps for each phase i(=1,2,3,4) for switching Go (see Supplementary Figure S1) is $8\times 10^{4}$. To achieve a constant temperature ensemble, velocity rescaling [34] was employed. The mass *m* for all amino acids was identical: m=10. For the Newtonian dynamics system, we investigated a 2-dimensional parameter space defined by temperature $T\times 10^{3}=2^{0},2^{1}\cdots 2^{20}\left[K\right]$ and the excluded volume interaction strength EVI$=2^{0},2^{1}\cdots 2^{11}$. For each combination of parameters (*T* and EVI) we conducted 10 trial simulations from the same initial structure (the 1994 complex structure) with different initial velocity conditions. See the supplemental text-S2 for the detailed of the investigation related to underdamped Langevin dynamics.

### 2.4. Definition of Success Rate for the Rotation of γ Subunit in F1-Motor

In CG-simulation, we observed the rotation angle θ(t) of γ subunit from the initial structure as shown in Figure 2a (See the supplemental text-S3 for the detailed definition of the rotation angle θ). Figure 2b shows an example of the time-series of the rotation angle θ. Because we changed the reference structure $X_{i}$ and potential $V_{\alpha 3\beta 3}\left(R|X_{i}\right)$ for each phase *i*(=1,2,3,4) according to switching Go-model scheme (Supplementary Figure S1), in an energy minimum state (T=0 K) the rotation angle θ of each phase *i* should ideally agree with (i−1)×120 (degree), named as the “Reference angle”. The reference angle increases in a step-wise manner as shown in Figure 2b. However, because of the thermal noise at the finite temperature *T* in each phase *i* the rotation angle θ(t) fluctuates and deviates from the reference angle stochastically. Furthermore, because of the influence of inertia (m=10.0) and friction (Γ), even after switching the reference structure $X_{i}$, the rotation angle cannot respond to changes in the reference angle instantaneously. A finite relaxation time (delay) is required to achieve a rotation angle of 120 degrees after potential switching.

Therefore we set the tolerance range for the F1-motor rotation angle θ as shown in Figure 2b: this area allows ± 60-degree deviation of the rotation angle from the reference and allows for a delay in angle response within the half time step in each phase after the switching potential. The goodness score of F1-motor rotation is defined as the ratio of the angle θs in the tolerance range during the whole trajectory. In this study, we defined a trial simulation with a goodness score of over 95% as a successful trial. The success rate is defined as the ratio of the number of successful trials out of 10 trials with different initial conditions for each parameter.

## 3. Results

### 3.1. Sampling Performances for F1-Motor Simulations Using Newtonian Dynamics

**Figure 3:**
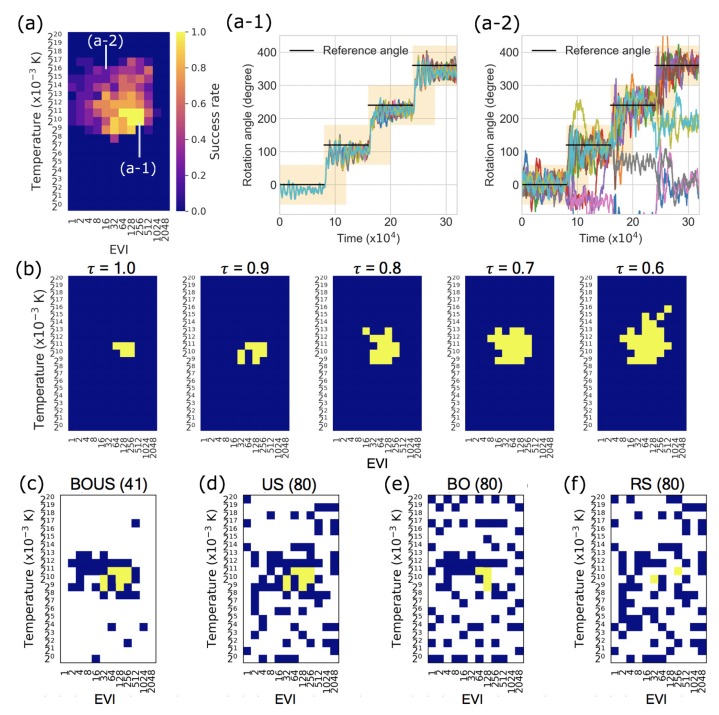
(**a**) Success rate distribution of F1-motor rotation based on Newtonian dynamics simulations in the parameter space. (a-1) and (a-2) show examples trajectories of F1-motor rotations in (**a**). The success rates were 1 and 0.2, respectively. (**b**) Regions of successful parameters changing the criteria (success threshold τ) to ensure success. The yellow and blue grids are “success” and “failure” parameters in each threshold τ, respectively. (**c**–**f**) are typical examples of successful parameter search using BOUS, US, BO, and RS. The numbers of sampled parameters were 41 for BOUS and 80 for US, BO, and random sampling (RS).

Figure 3a shows the success rate distribution of the F1-motor rotation based on Newtonian dynamics simulations for all parameters. Figure 3a-1,a-2 are examples of angle trajectories of F1-motor rotations in Figure 3a, and their success rates were 1 and 0.2, respectively. In this system, the rotation tended to be successful when the temperature was quite low, around 1 K, and when the EVI was as large as several tens to several hundreds of times of the default parameter. The region of successful parameter changes is shown in Figure 3b according to the criteria (Success threshold τ).

We applied the sampling algorithms BOUS, US, BO, and RS in Newtonian dynamics simulations. Figure 3c–f are typical examples of sampling results with the success threshold of 0.9 from the 10 same initial points using BOUS, US, BO, and RS, respectively. Each number in parentheses shows the number of sampled parameters. BOUS and BO began to search for successful parameters based on Bayesian optimization using the success rate information (Figure 3a), as the first 10 points did not contain successful parameters. BOUS switched to the US sampling immediately after finding the initial successful parameter at the 25th sampling and searched around the boundary between the successful and failed parameters. Finally, BOUS succeeded in searching for all (eight) successful parameters with only 41 samplings as shown in Figure 3c. In contrast, BO found the same successful parameter at the 25th sampling, but only four successful parameters were obtained at the 80th sampling, as shown in Figure 3e. The US succeeded in searching for all successful parameters with 80 samples (Figure 3d) because of intensive sampling around the boundary between the successful and failed parameters soon after finding the first successful point. In this trial, 64 points were required to find a successful parameter using RS. As a result, although 80 points were sampled in Figure 3f, only two successful parameters were obtained using RS.

**Figure 4:**
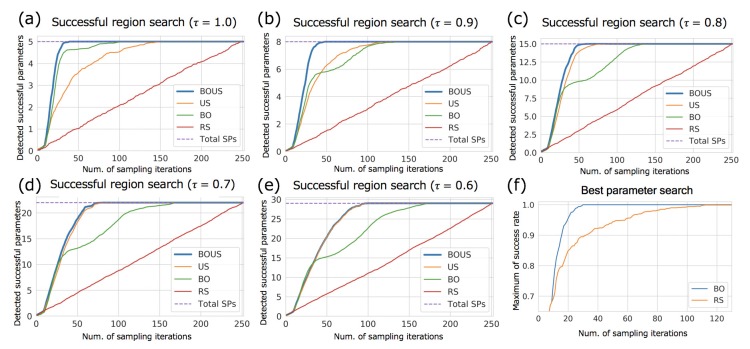
Performance of successful parameter search for Newtonian dynamics simulations using BOUS, US, BO, and RS. The success thresholds were 1.0 (**a**), 0.9 (**b**), 0.8 (**c**), 0.7 (**d**), and 0.6 (**e**), respectively. The dashed blue line shows the total number of successful parameters for each threshold. (**f**) shows the efficiency of the search for best parameter detection using BO and RS with τ=1.0.

To accurately evaluate the performances of the sampling methods, we calculated the performances of successful parameter search by randomly changing the initial points. Figure 4a–e show the numbers of detected successful parameters as functions of the number of sampling points for each success threshold. Here, the initial sampling number was fixed to 10. To evaluate the effect of different initial samplings, we repeated 100 trials using different initial points and averaged the results. We listed the number of iterations in the best and worst cases to find all successful parameters in Supplemental Table S1. As shown in Figure 4a–e, BOUS, US, and BO showed better performances compared to RS; particularly, BOUS surpassed the other machine learning-based approaches. For example, when the threshold τ = 1, BOUS identified all successful parameters with 15 iterations in the best case and with 40 iterations in the worst case as shown in Supplemental Table S1. The reason why the performance of BOUS was higher than that of US, particularly for larger thresholds, is considered to be that successful parameters can be searched quickly based on Bayesian optimization in the early stages of sampling. We showed the search performance to detect a successful parameter using BO and RS in Figure 4f. Each line is the average of 100 search trials with different initial parameters. The result showed that BO required only 30 samples to find a successful parameter even in the worst case, while more than 100 samples were needed by RS in some cases. For US, if there is only one phase, i.e., no successful cases have been found, the search by US will be closer to RS because no boundary is defined. The performance of US is considered to be inferior to BOUS, as time is required to find a successful parameter when the threshold is large. In contrast, when the threshold was less than 0.7, the performance of BOUS was nearly the same as that of US. This may be because when the threshold is small, the region of the successful parameter is large and can thus be immediately found by US. BO showed relatively high performances when the threshold was 1.0. In this case, the number of successful parameters was five, and BO could find these parameters during searching for the highest rate. On the other hand, it was confirmed that BO is not suitable for the exhaustive search of the successful parameters because the successful parameter is large when the threshold is small. We summarized the reduced performances of sampling iterations of BOUS, US, BO, and RS compared to exhaustive search in Table 1. The listed values of each sampling algorithm are the numbers of samplings required to detect all successful parameters with a probability of 95% or higher. The values in parentheses indicate the ratios of the number of calculations to the exhaustive search. From this result, the computational cost to identify the successful region can be reduced to 12.3% compared to exhaustive search by using BOUS when τ = 1.

### 3.2. Sampling Performance of F1-Motor Simulations Using Langevin Dynamics

**Figure 5:**
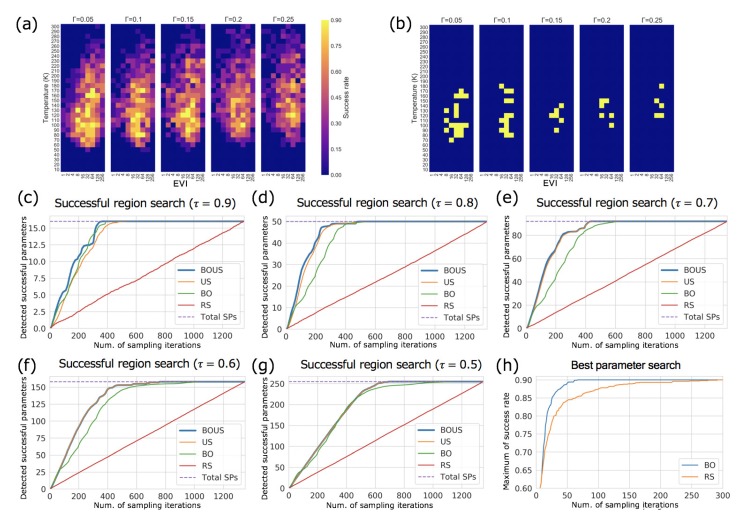
Results of Langevin dynamics simulations. (**a**) Success rate distribution in the parameter space. (**b**) Successful parameter region (yellow) with τ=0.8. (**c**–**g**) show performances of parameter region search using BOUS, US, BO, and RS. The success thresholds are 0.9 (**c**), 0.8 (**d**), 0.7 (**e**), 0.6 (**f**), and 0.5 (**g**), respectively. (**h**) shows the performance of BO and RS for the best parameter search.

We show the success rate distribution of all parameters based on Langevin dynamics simulations in Figure 5a, using two-dimensional maps with different Γs. The simulation results seem noisy compared to those obtained by Newtonian dynamics and the maximum success rate was 0.9. Successful parameters were found to exist over a relatively wide temperature range from 50 to over 200 K and a wide range of the excluded volume term from 8 to 256. Figure 5b shows the distribution of the successful parameter when the success threshold τ is 0.8. Distributions of the successful parameter with other values of threshold τ are shown in Supplemental Figure S2.

Figure 5c–g show the numbers of detected successful parameters as functions of the number of sampling points using BOUS, US, BO, and RS. The success threshold in Figure 5c–g were 0.9, 0.8, 0.7, 0.6, and 0.5, respectively. Each dashed line shows the number of successful parameters. The initial number of sampling trials was also fixed to 10. To evaluate the effect of different initial samplings, we also repeated 100 trials using different initial points. These results show that BOUS, US, and BO achieved better performance compared to RS, and BOUS was the best sampling method among them as Newtonian dynamics cases. Furthermore, the results of successful parameter searches by BO and RS are shown in Figure 5h. Each line is the average of 100 search trials with different initial parameters. This result shows that a successful parameter can be found efficiently by using BO, even in a relatively noisy distribution of success rate (Figure 5a) and in the three-dimensional parameter space. There was a tendency for the efficiency to deteriorate as the success threshold τ was reduced. This is thought to be mainly because the smaller the threshold τ value is, the larger the number of successful parameters and thus the larger the number of samples required to enumerate all successful parameters. We also summarized the reduction performance of sampling iterations of BOUS, US, BO and RS compared to exhaustive search in Supplemental Table S2. When the threshold is 0.8, the number of samplings can be reduced to 18.9% by using BOUS compared to the exhaustive search of the parameter space. These results confirmed that machine learning-based approaches were effective for F1-motor simulation with Langevin dynamics and the combination of BO and US (BOUS) enabled efficient identification of the successful region.

## 4. Discussion

By conducting Newtonian dynamics and under-damped Langevin dynamics simulations of a CG-model with a few specific parameters, the past works [3,32] (Koga’s work and CafeMol manual) showed that the F1 motor can make rotational motion: $T=4\times 10^{-3}$ K, $2\times 10^{-2}$ K, and EVI = 129.75 for Newtonian dynamics and T=100 K, EVI = 129.75, and Γ=0.05 for Langevin dynamics were presented as representative successful parameters. In contrast, in this study, by drawing phase diagrams as shown in Figure 3a,b for Newtonian dynamics and Figure 5a,b for Langevin dynamics, we elucidated that the F1 motor can reproduce the rotational motion with a high success rate over wider parameter areas than the (localized) specific parameters used in previous studies [3,32] (and an example in CafeMol manual). The time trajectories displayed in Supplemental Figure S3a,b for Newtonian dynamics show that even at a higher temperature T≃2.0∼8.2 (than $2\times 10^{-2}$) and lower EVI=16∼64 (than 129.75), the success rate for rotational motion of the F1 motor can be significantly high (0.8∼1). Similarly, the time trajectories for Langevin dynamics in Supplemental Figure S4b,c showed that even at a higher temperature T≥160 K and lower EVI ≤32∼64 than those in past work [32] (Figure S4a), a high success rate could be realized. Obtaining this kind of knowledge is one of the advantages of drawing a phase diagram in a wide parameter space of CG-model.

Clarifying the area of successful parameters will enable a detailed analysis of the dynamics and mechanisms to realize important functions of target biomolecules. For example, our results provide insight into the effect of friction Γ on the success rate from the phase diagram Figure 5a: while in the higher temperature area T≥280 K, the difference in success rates between low friction Γ=0.05 and high friction Γ=0.25 is not so significant, at the lower temperature T≤70 K, the success rate seems to be decreased with higher friction Γ. These tendencies may be apprehended from the simulated trajectories of the rotational angle in each parameter: at the lower temperature (T=70 K), compared to the rapid response for the smaller friction (Γ=0.05), the higher friction (Γ=0.25) caused a slower response and tended to fail rotational motion, as shown in Supplemental Figure S5. However, at the higher temperature T=280K, the fluctuation amplitude of rotation with lower friction (Γ=0.05) frequently exceeded the tolerance range of the angle, resulting in a lower success rate which is comparable to that obtained with high (Γ=0.25), as shown in Supplement Figure S6.

In our study, it seems that the temperature with a significant success ratio is relatively smaller than room temperature (∼300 K) in which the F1 motor can rotate in-vitro experiments. This probably comes from the switching go model: the immediate high activation energy of the whole system accompanied by switching potential may cause unstable rotation of gamma. We guess that the success ratio at higher temperatures can be improved by applying a multiple-basin model which can suppress activation energy.

Identifying the region of successful parameters for the CG-model will also provide information on the robustness of the biological function of the target molecule to environmental changes (temperature, ion strength and so on) and mutations. In the phase diagram, the range of successful EVI: [16:256] for Langevin dynamics (Figure 5b) and [8:128] for Newtonian dynamics with threshold τ=0.8 (Figure 3) is assumed to be related to the robustness in undergoing rotational motion of F1 against mutations in residues between γ and α3β3 subunits (as the EVI parameter should depend on the residues type in the corresponding area). Although in this study, the EVI parameter was set to a uniform value for all residues between γ and α3β3, a phase diagram with residue-dependent EVI parameters will be developed in our future studies.

To select the next parameter based on machine learning algorithms, computational costs (search time) are required for learning and selection. Supplemental Figure S7 shows the averaged search time of each algorithm at each sampling step in the Langevin dynamics simulations. These results show that the search time of BO was very short (approximately 10 s) compared to the simulation times. Furthermore, in the US, the search time was less than 1 s, and the search time decreased as the number of samplings increased. It is considered that label estimation, which is the most time-consuming step in US, converged quickly when the number of sampled points is large. Because BOUS initially uses BO, a relatively long search time is needed; however, after finding a successful parameter, this method switches to the US and the search time becomes very short. These search times are considered to be sufficiently short compared to time-consuming MD simulations.

In this study, we examined phase diagrams of parameter spaces with two phases, successful and failed. The phase diagram construction method based on US is also applicable in cases where there are more than two types of phases, as described in [22]. For example, in the case of the F1-motor system, because the substep (90 + 30) for 120-degree rotation was frequently observed at lower ATP concentrations in in-vitro experiments [35] there are three possibilities: 120-degree rotation at once (without substep), substep rotation (90 + 30 degree), and others (failure for rotation). It is considered effective to apply US simply or search for all phases using BO and then switch to US like BOUS, when there are more than two types of phases. As future work, we will apply our new methodology to other biological systems to evaluate the potential of our machine learning-based approach.

## 5. Conclusions

In this study, we showed that parameter sampling methods based on machine learning algorithms are effective for identifying the region of successful parameters. In particular, BOUS showed better performances compared to existing methods Bayesian optimization (BO) and Uncertainty Sampling (US), which is proposed to make a phase diagram efficiently. We confirmed that our methods were effective for F1-ATPase using different calculation methods, Newtonian and Langevin dynamics, and with different dimensions of parameters. We confirmed that the F1 motor can rotate successfully in a wider range of parameter space than previously reported. Basically, the machine learning-based approaches (BOUS, US, and BO) works if the success rate (success or failure) of a given parameter can be calculated in any simulation and modeling. Therefore, these methods can be widely applicable to studies of CG models that contain not only Go-model but also non-Go-model such as an elastic network model, as long as there is a successful area. Our published implementation can be used as an alternative for parameter determination approaches for biomolecule such as force matching [14] and fluctuating matching [15].

Our methods can be applied to other target systems and calculation methods, including CG-MD, all-atom MD simulation, and others, as long as the success rate can be defined. Especially, making the phase diagram of a specific CG model and biomolecule system with the proposed method would help to properly evaluate advantages and disadvantages such as applicable limits and bias of the CG model. In addition, it is possible to use the proposed methods even if the dimension of the parameter is four or more. However, BO becomes inefficient if the parameter dimension is too high; in this case, dimension reduction methods such as random embedding [36,37] would be effective.

## Figures and Tables

**Table 1:** Numbers of samplings required to detect all successful parameters with a probability of 95% or higher using BOUS, US, BO, and RS. SP indicates the number of successful parameters. The values in parentheses indicate the ratios of the number of calculations in the exhaustive search. The total number of parameter candidates was 252.

τ	SP	BOUS	US	BO	RS
1.0	5 (1.98%)	**31 (12.3%)**	115 (45.6%)	67 (26.6%)	237 (94.0%)
0.9	8 (3.17%)	**36 (14.3%)**	90 (35.7%)	100 (39.7%)	241 (95.6%)
0.8	15 (5.95%)	**45 (17.9%)**	56 (22.2%)	114 (45.2%)	242 (96.0%)
0.7	22 (8.73%)	**60 (23.8%)**	65 (25.8%)	129 (51.2%)	238 (94.4%)
0.6	29 (11.5%)	**80 (31.7%)**	82 (32.5%)	146 (57.9%)	241 (95.6%)

## Supplementary Materials

The following are available online at https://www.mdpi.com/2218-273X/10/3/482/s1.
