# Supplementary material for: Exploring Successful Parameter Region for Coarse-Grained Simulation of Biomolecules by Bayesian Optimization and Active Learning
Source: Biomolecules. 2020 Mar 21;10(3):482. doi: 10.3390/biom10030482 (PMC7175118; doi:10.3390/biom10030482)
Supplement: Supplementary file 1 [file biomolecules-10-00482-s001.pdf]

Supporting Information for "Exploring Successful Parameter Region for Coarse-Grained Simulation of Biomolecules by Bayesian Optimization and Active Learnin"

Ryo Kanada, Atsushi Tokushisa, Koji Tsuda, Yasushi Okuno and Kei Terayama

March 21, 2020

1 Supplemental Text-S1

1.1 Potential energy of F1-motor and simulation protocol

In this study, total potential energy for the F1-motor consists of four terms: $V = V_\gamma + V_{\alpha_3\beta_3} + V_{\alpha_3\beta_3-\gamma} + V_{anchor}$.

V_γ is the off-lattice Go model for the intra γ subunit, in which the reference structure of γ was determined in 1994 (1BMF) and all parameters were described in Koga's work. See the next subsection for the detailed information of the off-lattice Go model potential V_γ and parameters.

$V_{\alpha_3\beta_3}(R|X_i)$ is the switching Go model for the $\alpha_3\beta_3$ ring, in which R corresponds to the temporal structure and X_i corresponds to the reference structure based on the 1994 complex. To mimic ATP hydrolysis in our simulation, we switched the interaction potential $V_{\alpha_3\beta_3}(R|X_i)$ for the $\alpha_3\beta_3$ ring according to CG-simulation scheme as shown in Fig. S1.The entire simulation for 360-degree rotation of the γ subunit is divided into 4 phases. Starting from phase-1 , after the constant time steps are simulated in each phase (8×10^4 steps for Newtonian dynamics, 8×10^5 steps for Langevin dynamics), then interaction potential $V_{\alpha_3\beta_3}(R|X_i)$ is switched into the next phase:$V_{\alpha_3\beta_3}(R|X_{i+1})$. The transition from i to $i+1$ is assumed to be accompanied by ATP binding ($E \rightarrow TP$), ADP and Pi release ($DP \rightarrow E$), and ATP hydrolysis ($TP \rightarrow E$) in each $\alpha\beta$ subunit. The parameters for $V_{\alpha_3\beta_3}(R|X_i)$ are same as those used by Koga's work. (See the next subsection for detailed information of parameters of $V_{\alpha_3\beta_3}$).

$V_{\alpha_3\beta_3-\gamma}$ is the excluded volume interaction (EVI) between γ and $\alpha_3\beta_3$ subunit: which is defined as

$$V_{\alpha_3\beta_3-\gamma} = \sum_{i\in\alpha_3\beta_3, j\in\gamma} EVI \times \epsilon(C/r_{ij})^{12}. \quad (1)$$

In this study C was 4.0 Å, ϵ was 0.36 kcal/mol, EVI was set to various values. Concretely, EVI is power of 2: EVI =1,2,4 $\ldots 2^N$; N was 8 for Langevin dynamics and 11 for Newtonian dynamics (EVI= 128 $\simeq$ 129.75 corresponding to the case studied in Koga's work).

V_{anchor} is introduced to maintain the F1-atpase complex system on a plane. This potential is defined as follows:

$$V_{anchor} = \sum_{i\in\beta_1 9,\beta_2 9,\beta_3 9,\gamma 272} \begin{cases} k(L_i - L_i, 0)^2 & (L_i \geq L_i, 0) \\ 0 & (otherwise), \end{cases} \quad (2)$$

where k is 0.18 kcal/mol/Å^2, L_i is the distance of i-th residues from initial position, and $L_i, 0$ is 1.5 Å for $\beta_1 9$, $\beta_2 9$, and $\beta_3 9$ and 2.0 Å for γ 272 which were described by Koga's work.

1.2 Energy function of off-lattice Go model and model parameters

In this study, particulary for V_γ (interaction of the intra-γ subunit) and $V_{\alpha 3\beta 3}$ (interaction of $\alpha_3\beta_3$ ring) the following Clementi's type of the C^α Go potential are applied:

$$\begin{aligned} V(\mathbf{R}|\mathbf{X}) = & \sum_{ibd} k_b(b_{ibd} - b_{ibd,X})^2 + \sum_{iba} k_\theta(\theta_{iba} - \theta_{iba,X})^2 \\ + & \sum_{idih} \{k_\phi^{(1)}\left[1 - cos(\phi_{iba} - \phi_{iba,X})\right] + k_\phi^{(3)}\left[1 - cos(\phi_{iba} - \phi_{iba,X})\right]\} \\ + & \sum_{i<j-3}^{natcontact} \epsilon\left[5(\frac{r_{ij,X}}{r_{ij}})^{12} - 6(\frac{r_{ij,X}}{r_{ij}})^{10}\right] \\ + & \sum_{i<j-3}^{non-native} \epsilon(\frac{C}{r_{ij}})^{12}, \end{aligned}$$

where $\mathbf{R}$ represents the Cartesian coordinates, and $\mathbf{X}$ signifies the native structure; in switching Go-model reference structure $\mathbf{X}$ is switched according to scheme as shown in Fig. S1. In the above equation, the first term maintains the length of the virtual covalent bond; b_{ibd} is the length of the i-th virtual bond between the i-th and $i-1$-th amino acids. The second and third terms indicate the local bond angle and dihedral angle interactions: θ_i and ϕ_i are virtual bond angle between $i-1$-th and i-th bond and around i-bond respectively. The fourth term is the native contact pairs, which are defined as follows: when one of the non-hydrogen atoms in the i-th amino acid is within a distance of 6.5 Å from any non-hydrogen atom in the j-th amino acid in the native structure, the i-th and j-th amino acids are regarded as forming a native contact pair. The last term reflects excluded volume interactions (EVIs) between non-native contact pairs. Parameters with the subscript X are constants, whose values are taken from the the native structure $\mathbf{X}$. We use the following values for interaction parameters throughout the present study: $k_b = 100.0$, $k_\theta = 20.0$, $k_\phi^{(1)} = 1.0$, $k_\phi^{(3)} = 0.5$, $\epsilon = 0.36$, and $C = 4.0$ Å, which are the same as those used by Koga and Takada.

2 Supplemental Text-S2

2.1 Detailed of under-damped Langevin dynamics and sampled parameter space

For time integration of underdamped Langevin dynamics, we used the following equation with a step size of $dt = 0.2$:

$$m_i \frac{d^2\mathbf{r_i}}{dt^2} = -m_i \Gamma \frac{d\mathbf{r_i}}{dt} - \frac{dV}{d\mathbf{r_i}} + m_i \xi_i, \tag{3}$$

where m_i ($= 10.0$) is the mass of each amino acid, V is the total system potential energy function, Γ_i is the friction coefficient, and ξ_i is the random force in solution environment. The time steps of each phase $i(= 1, 2, 3, 4)$ for switching Go (see Fig. S1) is 8×10^5. The random force is defined by a white and Gaussian noise that satisfies the following fluctuation theorems:

$$\langle \xi_i \rangle = 0, \tag{4}$$

$$\langle \xi_i(t) \xi_j(t') \rangle = 2 \frac{\Gamma_i k_B T}{m_i} \delta(t - t') \delta_{ij}, \tag{5}$$

where the bracket indicates the ensemble average, k_B is the Boltzmann constant and T is the temperature. For the underdamped Langevin dynamics system we investigated the 3-dimensional parameter space defined by temperature $T = 10, 20, 30 \cdots 300[K]$, excluded volume interaction strength EVI$= 2^0, 2^1, 2^2 \cdots 2^8$, and friction coefficient $\Gamma = 0.05, 0.1, 0.15, 0.2, 0.25$. For each combination of parameters (T, EVI, and Γ) we conducted 10-trial simulations from the same initial structure (1994 complex structure) with different random noise sets $\{\xi\}$.

Here we have to note that the friction coefficient $\Gamma \leq 0.25$ is much smaller than $\Gamma \simeq 45$ which estimated by taken account of the viscosity of water 8.0×10^{-4} kg/(m s) in the experimental environment of the motor. In this study we applied the relatively lower friction coefficients Γ because we want to reproduce qualitative rotational motion of gamma-subunit of F1 smoothly with realistic simulation time steps.

3 Supplemental Text-S3

3.1 Definition of rotation angle $\theta(t)$ of γ subunit in F1 motor

In our simulation system, the long axis of γ-subunit is nearly perpendicular to the plane defined by the three β 9 C_α positions anchored by the positional constrained potential V_{anchor} and passes the center of the triangle made by the three C_α atoms. The counterclockwise rotation of the γ-subunit from the initial structure is monitored by a vector $\mathbf{\Omega}_{pro}(t)$ which is defined as the projected vector of $\mathbf{\Omega}(t)$ on the plane which is defined as three anchored points (β 9).

$$\mathbf{\Omega}(t) = \mathbf{r}_{\gamma\mathbf{58}}(t) - \mathbf{r}_{\gamma\mathbf{78}}(t), \tag{6}$$

where $\mathbf{r}(t)$ is the position of i-th C_α at time t. The rotation angle $\theta(t)$ is defined by the angle between $\mathbf{\Omega}_{pro}(t)$ and corresponding vector in the initial structure (1994 complex structure).

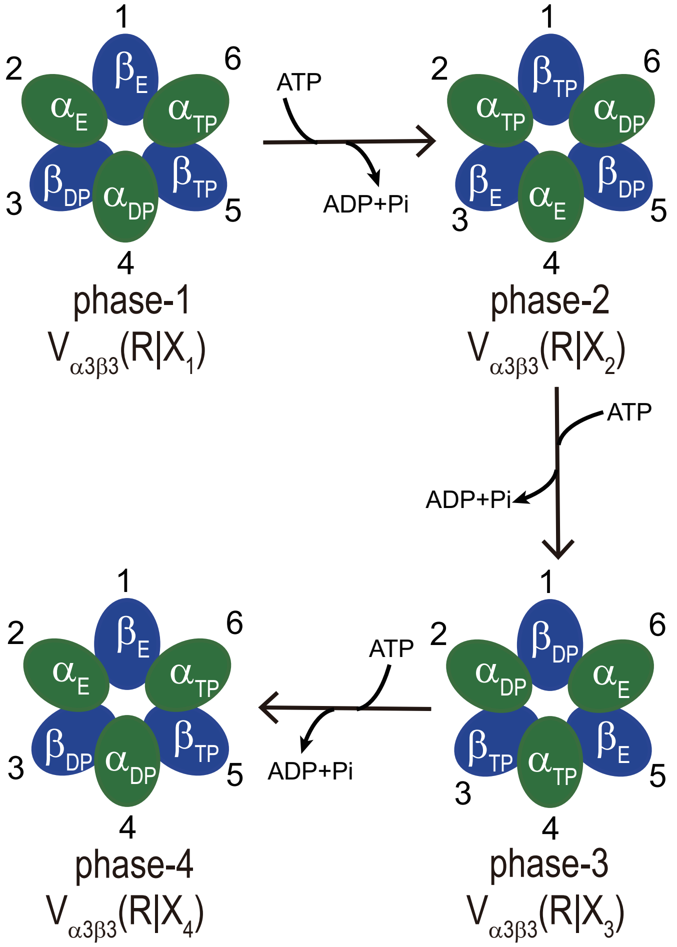

Figure S1: CG-simulation scheme. The ellipsoids (blue and green) correspond to the α and β subunits, respectively. The "TP", "DP", and "E" are the ATP-bound, ADP-bound, and nucleotidefree states, respectively. The entire simulation cycle for 360-degree rotation is divided into 4 phases. In each phase i $(= 1, 2, 3, 4)$, $V_{\alpha 3\beta 3}(R|X_i)$ indicate the potential of $\alpha_3\beta_3$ ring for which the reference structure X_i is based on the 1994 structure. The transition $i \to i+1$ phases are accompanied by ATP binding $(E \to TP)$, ADP and Pi release $(DP \to E)$, and ATP hydrolysis $(TP \to E)$ in each $\alpha\beta$ subunit. Each phase is simulated for the constant time steps (8×10^5 steps for Langevin dynamics, 8×10^4 for Newtonian dynamics).

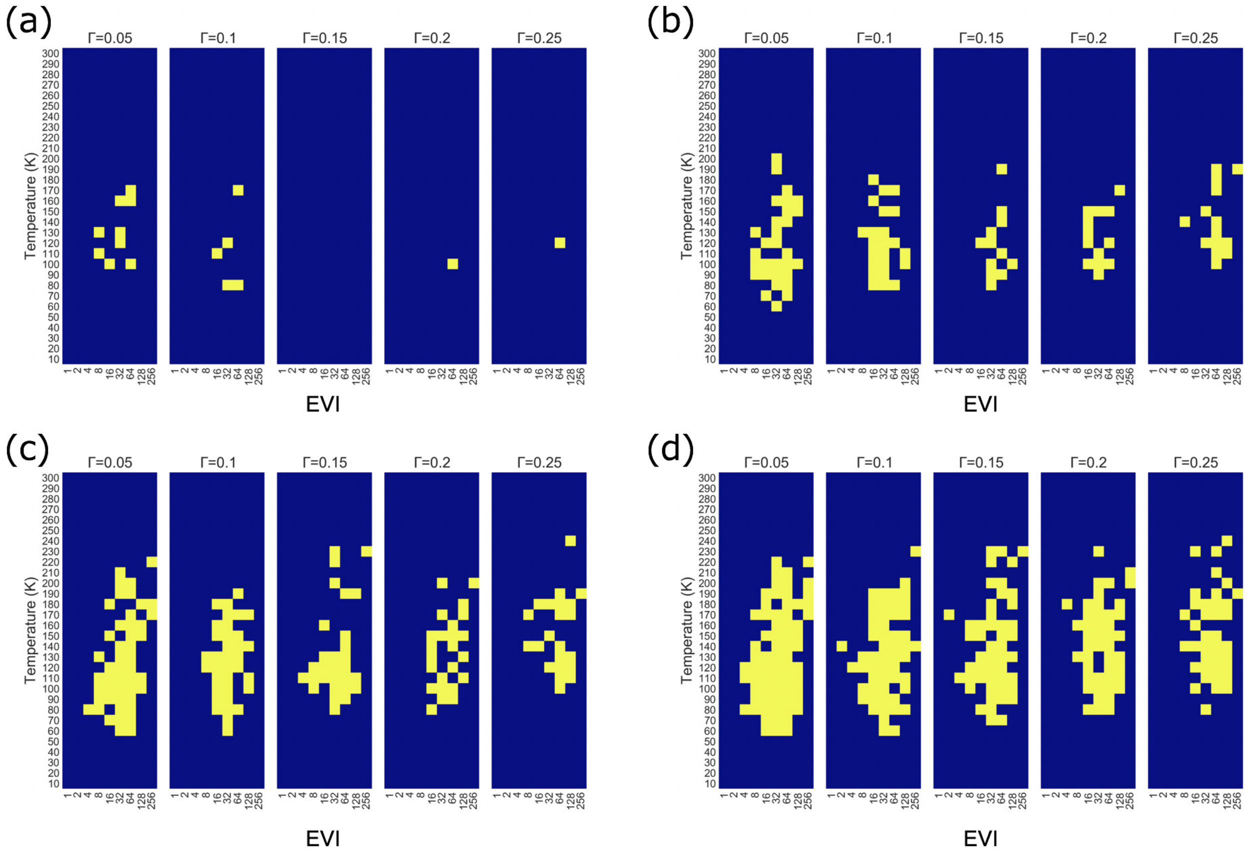

Figure S2: Distributions of the successful parameter with different threshold τ for Langevin dynamics simulations. The yellow and blue grids indicate "success" and "failure" parameters, respectively. The thresholds are 0.9 (a), 0.7 (b), 0.6 (c), and 0.5 (d).

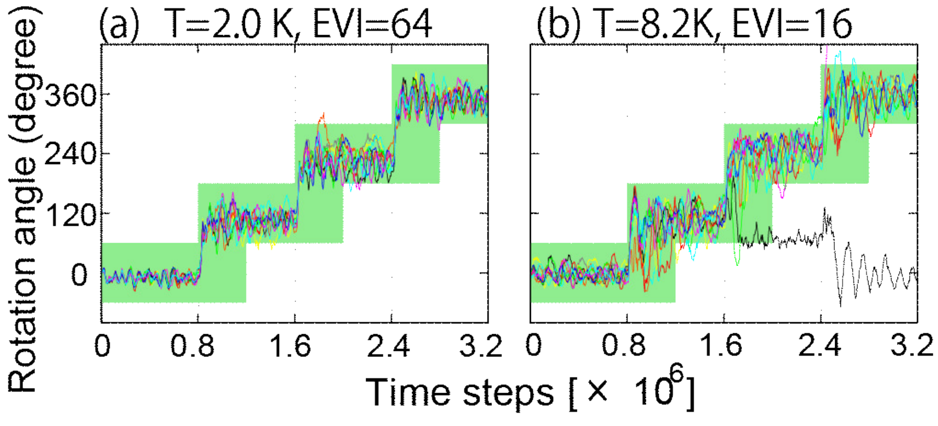

Figure S3: Time trajectory of rotational angle by Newtonian dynamics with specific parameters.

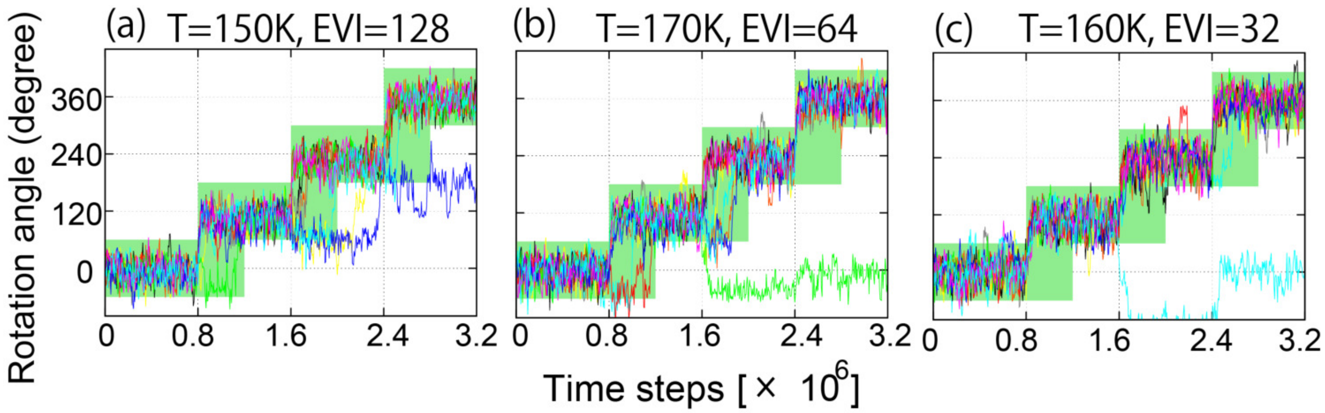

Figure S4: Time trajectory of rotational angle by Langevin dynamics with specific parameters.

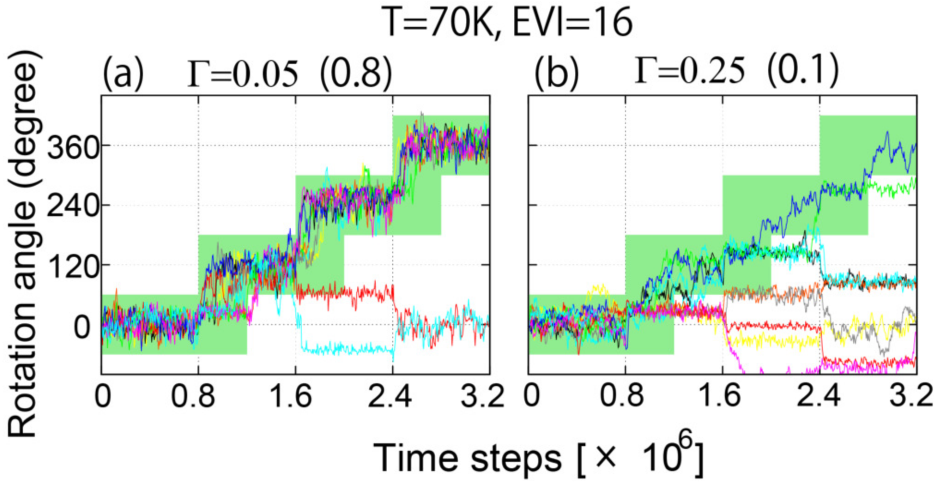

Figure S5: Time trajectory of rotational angle by Langevin dynamics at lower temperature $T = 70K$. The values in parentheses indicate the success rates.

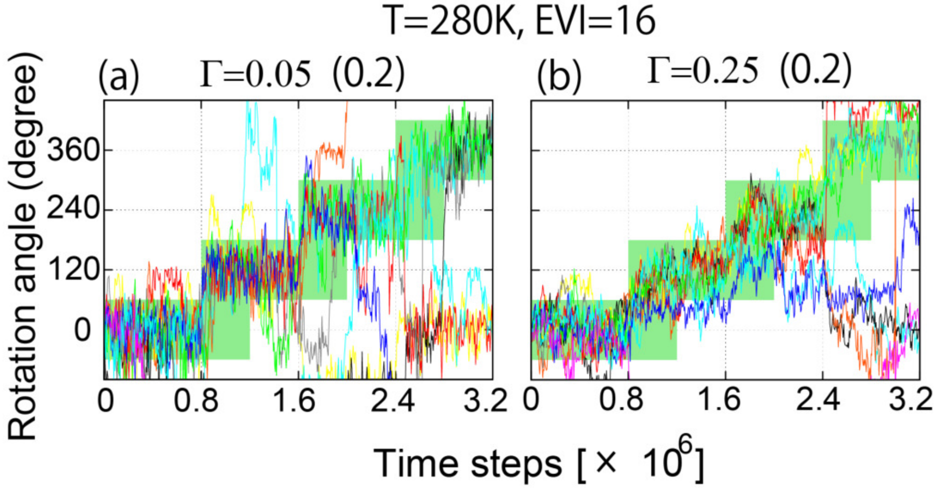

Figure S6: Time trajectory of rotational angle by Langevin dynamics at higher temperature $T = 280K$. The values in parentheses indicate the success rates.

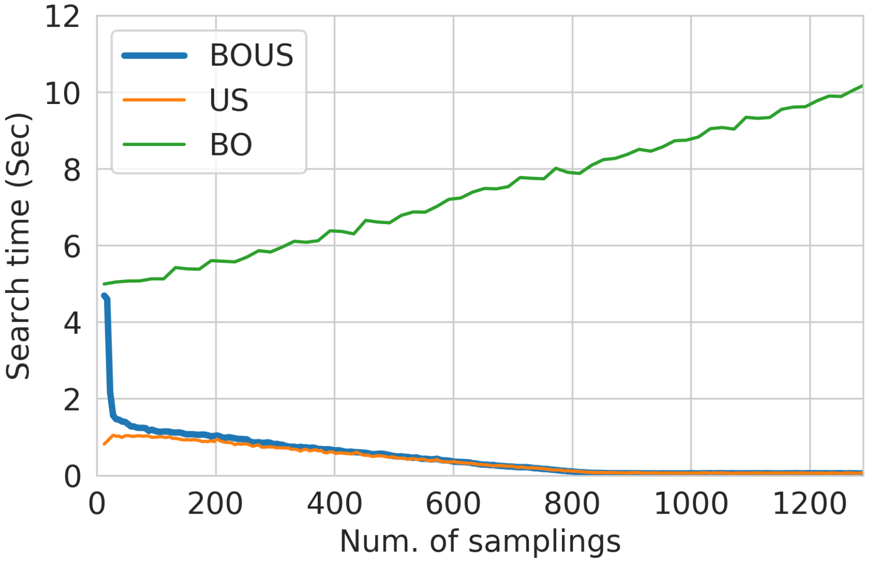

Figure S7: Search times to select the next parameter by BOUS, US, and BO in Langevin dynamics simulation.

Table S1: **Numbers of samplings required to detect all successful parameters in best and worst cases using BOUS, US, BO, and RS for Newtonian dynamics simulations. SP indicates the number of successful parameters. The total number of parameter candidates was 252.**

		BOUS		US		BO		RS	
τ	SP	best	worst	best	worst	best	worst	best	worst
1.0	5	15	40	16	151	16	102	93	250
0.9	8	24	49	21	122	23	135	110	252
0.8	15	41	63	43	92	81	145	191	252
0.7	22	55	82	57	107	87	176	213	252
0.6	29	75	101	78	107	115	176	211	252

Table S2: **Numbers of samplings required to detect all successful parameters with a probability of 95% or higher using BOUS, US, BO, and RS. The values in parentheses indicate the ratios of the number of calculations to the exhaustive search. The total number of parameter candidates was 1320.**

τ	Num. of successful parameters	BOUS	US	BO	RS
0.9	16 (1.21%)	**324 (24.5%)**	386 (29.2%)	341 (25.8%)	1279 (96.7%)
0.8	50 (3.79%)	**250 (18.9%)**	285 (21.6%)	383 (29.0%)	1290 (97.7%)
0.7	92 (6.97%)	**397 (30.1%)**	**397 (30.1%)**	454 (34.4%)	1285 (97.3%)
0.6	158 (12.0%)	**440 (33.3%)**	**440 (33.3%)**	563 (42.7%)	1281 (97.0%)
0.5	255 (19.3%)	**576 (43.6%)**	577 (43.7%)	649 (49.2%)	1285 (97.3%)
